# Comparative Transcriptome Analyses of the Developmental Stages of *Taenia multiceps*

**DOI:** 10.3389/fvets.2021.677045

**Published:** 2021-07-05

**Authors:** Wen-Hui Li, Yang Yang, Nian-Zhang Zhang, Jian-Kui Wang, Yin-Ju Liu, Li Li, Hong-Bin Yan, Wan-Zhong Jia, Baoquan Fu

**Affiliations:** ^1^State Key Laboratory of Veterinary Etiological Biology, Key Laboratory of Veterinary Parasitology of Gansu Province, Lanzhou Veterinary Research Institute, Chinese Academy of Agricultural Science, Lanzhou, China; ^2^Wuhan Animal Disease Prevention and Control Center, Wuhan, China; ^3^Center for Animal Disease Control and Prevention of Qilihe District, Lanzhou, China; ^4^Jiangsu Co-innovation Center for Prevention and Control of Important Animal Infectious Diseases and Zoonoses, Yangzhou, China

**Keywords:** comparative transcriptome, developmental stages, *Coenurus cerebralis*, differentially expressed gene, *Taenia multiceps*

## Abstract

Cerebral coenurosis, caused by the larvae of *Taenia multiceps* (*Coenurus cerebralis)*, is a fatal central nervous system disease in sheep and other herbivores and occasionally humans. Comparative transcriptomic profiles of the developmental stages of the parasite remain unknown. In this study, RNA sequencing was used to determine the transcriptome profiles of different stages of the life cycle of *T. multiceps*, including Oncosphere, *Coenurus cerebralis* (Pro with Cyst), and Adult (Adu), as well as scolex-neck proglottids (Snp), immature–mature proglottids (Imp), and gravid proglottids (Grp) of the adult stage. A total of 42.6 Gb (average 6.1 Gb) Illumina pair-end reads with a 125-bp read length were generated for seven samples. The total number of differentially expressed genes (DEGs) in the various life stages ranged from 2,577 to 3,879; however, for the tissues of the adult worm, the range was from 1,229 to 1,939. Kyoto Encyclopedia of Genes and Genomes analysis showed that the DEGs mainly participated in cellular and metabolic processes, binding and catalytic activity, genetic information processing, and environmental information processing. In addition, a large number of genes related to development and parasite–host interaction were identified. Quantitative reverse transcription-polymerase chain reaction confirmed that the levels of 28 selected DEGs were consistent with those determined using RNA sequencing. The present study provides insights into the mechanisms of the development and parasitic life of *T. multiceps*.

## Keypoints

- A total of 42.6 Gb (average 6.1 Gb) Illumina pair-end reads with a 125-bp read length were generated for seven samples from *T. multiceps*.
- The total number of DEGs in the various life stages of *T. multiceps* ranged from 2,577 to 3,879; however, for the tissues of the adult worm, the range was from 1,229 to 1,939.
- Some differentially expressed genes are involved in the development of *T. multiceps* and the parasite–host interaction.

## Introduction

*Taenia multiceps* is a tapeworm that inhabits the small intestine of dogs and other canids. The larval stage (known as *Coenurus cerebralis*) with a fluid-filled cyst is usually found in the nervous system of the intermediate host and often causes encephalitis and neurological symptoms in infected hosts, and sometimes even death (1, 2). Definitive hosts excrete egg-containing feces into the environment; when intermediate herbivorous hosts ingest these eggs during grazing, oncospheres spread from the eggs. Furthermore, through blood circulation, they reach various locations, including the central nervous system, where they could develop into mature Coenurus cysts of the larval stage within 2–3 months (3). The life cycle of *T. multiceps* is completed after the scoleces contained within the coenurus are ingested by the definitive host. The disease may last several months and is fatal (4, 5). *T. multiceps* infection in sheep results in significant economic losses in many parts of the world, predominantly in the developing countries of Africa, Europe, and Southeast Asia (1, 6, 7). Cases of human infections due to accidental ingestion of *T. multiceps* eggs have also been reported frequently and cause serious pathological conditions (8–12). Cestode development is well-known to involve complex morphological and physiological changes associated with changes in the host species (13, 14). Strobilation development (generation of serially repeated reproductive organs) is an interesting but difficult study subject, as it is difficult to obtain the parasites and maintain them in experimental hosts or *in vitro* (15). Recently, the genome of *T. multiceps* was sequenced to understand its biology and evolution (16). Extensive transcriptome analysis of *T. multiceps* larvae (17) and adults (18) provides substantial and useful information. Additionally, comparative transcriptomic analysis can identify key genes associated with the development, structure, and storage of carbohydrates, thereby contributing to the understanding of the biology of organisms (19–21). However, the mechanisms underlying the development of *T. multiceps* from larva to adult remain unclear. In the present study, comparative transcriptome analyses of the developmental stages of *T. multiceps* were performed to obtain important insights into the molecular basis of biological processes involved in worm development and reproduction.

## Materials and Methods

### Sample Collection

(i) The larvae of *T. multiceps* (named *Coenurus cerebralis*) were obtained from the brains of naturally infected sheep at a local slaughterhouse in Gansu province, China. Freshly separated *Coenurus cerebralis* were washed repeatedly in sterile phosphate-buffered saline (PBS, pH 7.4) to remove the host components. Protoscolices (Pro) were then separated from the cyst wall (cyst) of *Coenurus cerebralis*. (ii) Adult *T. multiceps* (Adu) were collected from experimentally infected dogs at our laboratory animal center. The dog infection experiment was performed according to a previously reported protocol (18, 22). In brief, two 4-month-old parasite-free beagle dogs were infected orally with 40 *T. multiceps* protoscolices. When gravid proglottids (Grp) were found in the feces, the infected dogs were exsanguinated, and the worms were collected and washed. Thereafter, scolex-neck proglottids (Snp), immature–mature proglottids (Imp), and Grp were separated from each adult based on the proglottid characteristics. (iii) For the isolation of oncospheres, gravid tapeworm segments were obtained from the above collected adult worms, which were then homogenized, filtered, washed, and purified by Percoll density-gradient separation. Finally, oncospheres (Onc) were hatched according to the procedure described previously (23, 24). To prepare tissue samples from adult *T. multiceps*, intact adults were segmented into three parts: Snp, Imp, and Grp based on their proglottid characteristics. All prepared samples were washed with sterile PBS, preserved in five volumes of RNA-later (TaKaRa, Japan), and then immediately stored in liquid nitrogen at −80°C for RNA sequencing (RNA-seq) analysis.

### Total RNA Isolation, Qualification, and Transcriptomic Library Construction

Total RNA was extracted from each sample using TRIzol Reagent (Invitrogen, USA), and contaminating genomic DNA was removed by treatment with RNase-free DNase I (Takara, Japan). RNA degradation and contamination were monitored on 1% agarose gels, and RNA integrity was assessed using an RNA Nano 6000 Assay Kit and Bioanalyzer 2100 system (Agilent Technologies, USA). RNA with an RNA integrity number (RIN) > 7.0 was considered to be of high enough quality for transcriptomic library construction and RNA sequencing, according to the manufacturer's instructions. Subsequently, mRNA was isolated from total RNA, cut into fragments, and reverse-transcribed into first-strand cDNA using random hexamers. Briefly, double-stranded cDNA was synthesized and purified, and the ends were repaired. Finally, RNA sequencing libraries were constructed using the Illumina TruSeq RNA Sample Preparation Kit (Illumina, San Diego, CA, USA), according to the manufacturer's instructions. Library quality was assessed using an Agilent Bioanalyzer 2100 system (Agilent Technologies).

### Transcriptome Sequencing and Data Analysis

Seven transcriptomic libraries were pooled and sequenced using the Illumina HiSeq 2000 platform (Illumina, San Diego, CA, USA). Raw reads in fastq format were first processed through in-house Perl scripts to obtain clean reads by discarding reads containing adapters, reads containing poly-N, and low-quality reads. Finally, high-quality reads were generated after RNA-seq and filtering using the FastQC software (25). The filtered reads were then aligned to the draft of the *T. multiceps* genome sequence, the indexes of Burrows–Wheeler were constructed using Bowtie2 in default parameters (26), and the clean data from seven RNA-seq libraries were mapped to the assembled genome of *T. multiceps* using the BWT-MEM software.

To assess the differentially expressed genes (DEGs) in each life-cycle stage and tissue, transcript FPKM values from a replicate were plotted against transcript FPKM values from another replicate using cutflinks (27). The false discovery rate (FDR) was used to determine the threshold *P*-values in multiple tests for comparing the differential expression. We used |log_2_ (FPKM_sampe1_/FPKM_sampe2_)| ≥ 1 and FDR ≤ 0.001 as the threshold to judge the significance of DEGs.

### Functional Classification of DEGs

Functional annotation of DEGs was performed using the UniProt database and BLASTP v2.3.0+ (28) and InterProScan (29) analyses. Gene ontology (GO) enrichment analysis of DEGs was performed using the GOseq R package (v1.10.0).

### Data Access

The RNA-seq data from this study have been deposited in NCBI Sequence Read Archive (SRA accession number PRJNA307624) and are accessible through accession numbers SRX2357663, SRX2357664, SRX2357665, SRX2357666, SRX2357667, SRX2357668, and SRX2357669.

### Quantitative Amplification to Verify DEGs Identified Using RNA-Seq

qPCR assay was performed to validate the DEGs identified using RNA-seq. Twenty-eight DEGs were selected and investigated using qRT-PCR at the transcriptional level. The β-tubulin gene (Tmu007343) was used as an internal control. All primers were designed using Primer Premier 5.0 and synthesized by Sangon (Shanghai, China) (Table 1). Total RNA was isolated as described above for the samples from protoscolices (Pro), cyst wall (Cyst), adult (Adu), scolex and neck proglottids (Snp), Imp, and Grp. cDNA was synthesized using the PrimeScript RT Reagent Kit with gDNA Eraser (TaKaRa). The qRT-PCR was performed on an ABI Stepone plus real-time PCR Detection System following the manufacturer's protocol for SG Fast qPCR Master Mix (High Rox) (2X) (BBI) (ABI). The PCR conditions were as follows: an initial denaturation step at 95°C for 3 min, followed by 45 cycles of denaturation at 95°C for 7 s, annealing at 57°C for 10 s, and extension at 72°C for 15 s. Relative quantification of all selected genes was performed using the 2^−ΔΔCT^ method.

**Table 1:** qRT-PCR primers of differentially expressed genes.

**Gene ID**	**Forward primer sequence (5^**′**^-3^**′**^)**	**Reverse primer sequence (5^**′**^-3^**′**^)**	**Gene annotation**
Tmu003811	TAGTAGGAAGAACGCCAAGAGC	TGACACTGCCTCCAACGTAACT	Oncosphere protein Tso31b [*Taenia solium*]
Tmu005434	CACAGTCTTATGGCTTTGCG	TCTTGCGTGGGTCTATTGC	Oncosphere protein Tm18 [*Taenia multiceps*]
Tmu012228	CAGTCAGCGTGGACAATGGA	TGTTTGCTTGCGAAGGATTG	Oncosphere protein Tm18 [*Taenia multiceps*]
Tmu007899	TCTCAGTGTCCGCAAGTCCA	GATTCAGCTCAGTCTGCCATG	Oncosphere protein Tm16 [*Taenia multiceps*]
Tmu005154	CGTGCCTACATTGTGCTTCTT	TTGCTTTCCAACTGGGTCAT	8 kDa glycoprotein [*Taenia multiceps*]
Tmu005158	CGTGCCTACATTGTGCTTTTC	CCAGTCCTTTGCCAGTTGAG	8 kDa glycoprotein[*Taenia hydatigena*]
Tmu005823	GGAAGACGATGGAAAGAGCAC	CTTCTGGCTTCGGACACTGTT	8 kDa glycoprotein[*Taenia hydatigena*]
Tmu003810	TGACTTCCAACCGCAACAA	CGTCGCAGTCACAAGGTATG	45m oncosphere protein [*Taenia multiceps*]
Tmu003173	CGGAGAGCAAGTTCAAGACC	GCTGCCTTAACGTAGGTTCG	Cytosolic fatty acid binding protein [*Taenia solium*]
Tmu012421	GAGGTCGTTGTGTCCCATTCTA	GCCTTCTTTACCCTTCTTGGAC	Hypothetical protein Y032_0068g222 [*Ancylostoma ceylanicum*]
Tmu000409	TGGAGCGGTTGTCATTGTG	TGGTGTTTGAACTGAAGGAGG	Tetraspanin [*Echinococcus multilocularis*]
Tmu004894	GGCGCTGATTCATACCCTAA	AGCATTGGGACAGTGGACTC	Putative insulin-like protein [*Taenia solium*]
Tmu004333	ACCTCCACTACCTCACCATCACT	CACTCCATAAGCCCACCAATC	cAMP dependent protein kinase catalytic subunit [*Echinococcus granulosus*]
Tmu006091	TCAAGAGGCTCTCGTTCGAT	TGAAGGGGAAGCAATTTTTG	Kunitz: Bovine pancreatic trypsin inhibitor [*Echinococcus granulosus*]
Tmu003193	CGAGCGGTATCAAAGGTAAGA	AGCCTCAAGGGATGGAACA	Glutamate dehydrogenase 2 [*Echinococcus granulosus*]
Tmu011357	CATCGTCTGAAGTGGTGGTGT	CTGAAATCGAACGCTCATAATC	T box transcription factor TBX6 [*Echinococcus multilocularis*]
Tmu012976	AAGGGAAGGAGGGTGTAGAAAC	TGTAATACCTGCTGACGACGATA	T box transcription factor TBX6 [*Echinococcus multilocularis*]
Tmu007808	ATGACGAGCAATACTTGTGGG	TGATAACTTCTTTGATGCGACG	Heat shock protein HSP 90-alpha [*Echinococcus granulosus*]
Tmu005042	CATTGTCGTCCACTCCTTTCC	TGACTGGCTCGTTGATTTCG	Fushi tarazu Antennapedia Ultrabithorax abdominal A [*Echinococcus granulosus*]
Tmu003765	GTCAACCCAGCGTACGATCT	CGAGGATGCCTTATCCTGTC	Aquaporin-9 [*Echinococcus granulosus*]
Tmu007712	GGTGGAATTGGAGGCTGATA	AACCGCCTCCATTTCCAT	Unknown
Tmu010856	TCTGACCATATTGTGAGCCCTT	AGAAAGATGGAGTGTGGAAGTTG	Unknown
Tmu010698	GACAACCAGCAAGAAAGCAATC	GCCCTCGAACAATGAATCAAC	Heat shock cognate protein[*Echinococcus granulosus*]
Tmu012864	AAAGAAGCGGGCAGAAATG	AACCTTCGGCTTTCCTTCC	Heat shock cognate protein[*Echinococcus granulosus*]
Tmu003182	AGCAGCAGGTTAGCAAGGAC	GCAAATCAGCTCATCGGTCT	Cytosolic fatty acid binding protein [*Taenia solium*]
Tmu005664	CGATCAACCCATTCAGATTTAC	GACACCGTTCTTCAAGTTACCA	cAMP dependent protein kinase catalytic subunit [*Hymenolepis microstoma*]
Tmu010513	TTGGTGGGCTGTTGGTGTT	TGGCTTTGGATGTCCGCTA	cAMP dependent protein kinase catalytic subunit [*Echinococcus multilocularis*]
Tmu007343 (internal control gene)	ACTCCGTGGTTCCTTCTCCT	CCAGACATTGTGGCACTGAC	Beta-tubulin

## Results

### Summary of RNA-Seq Data and Transcript Assembly

The seven RNA samples from the developmental stages of *T. multiceps* had prominent 18S and 28S ribosomal peaks on agarose gels (data not shown). All RNA samples possessed high integrity and purity with RINs>7.0, according to Bioanalyzer 2100 analysis, and were considered of sufficient quality for further experiments. Raw read sequences were filtered to discard adaptors and low-quality reads (error rate > 0.001) from all seven RNA-seq libraries. The clean data from seven RNA-seq libraries with a matching ratio >97% were mapped to the genome of *T. multiceps* (Table 2). Overall, a total of 42.6 Gb (average 6.1 Gb) Illumina pair-end reads with a 125-bp read length were generated for seven samples, including oncospheres, protoscolices and cyst wall, Snp, Imp, and Grp. A total of 8,208 to 10,498 predicted genes in all samples with FPKM value > 1, 4,396–6,652 genes with FPKM value >10, 752 to 1, and 290 genes with FPKM value >10 were detected (Table 3).

**Table 2:** RNA-seq reads for transcriptomes aligned to the assembled genome of *T. multiceps*.

**Sample**	**Average GC%**	**Raw reads**	**Clean reads**	**Ratio of clean reads**	**Matching reads**	**Matching ratio**
Onc	48.5	51,022,862	44,815,122	87.83%	43,922,159	98.0%
Pro	47.5	51,802,274	45,942,166	88.69%	44,568,458	97.0%
Cyst	47.0	47,781,894	41,028,226	85.87%	40,039,080	97.6%
Adu	48.5	54,360,816	49,654,526	91.34%	49,152,028	99.0%
Snp	50.0	56,183,654	47,750,165	84.99%	46,935,802	98.3%
Imp	49.0	49,695,832	46,823,629	94.22%	46,338,444	99.0%
Grp	49.0	54,779,876	47,353,255	86.44%	46,721,666	98.7%

*Onc, oncospheres; Pro, protoscolices; Cyst, cyst wall; Adu, adult; Snp, scolex-neck proglottids of adult; Imp, immature-mature proglottids; Grp, gravid proglottids of adult*.

**Table 3:** Statistics of expression genes in seven RNA-seq samples with FPKM > 1, FPKM > 10, and FPKM > 100.

**Sample**	**No. of genes**		
	**FPKM > 1**	**FPKM > 10**	**FPKM > 100**
Onc	8,208	5,545	1,197
Pro	9,169	6,347	1,248
Cyst	8,318	4,396	752
Adu	10,405	6,572	1,290
Snp	8,729	5,609	1,106
Imp	10,498	6,652	1,255
Grp	8,724	5,228	1,153
Total	11,317	8,170	2,218

*FPKM means fragments per kilobase of exon per million fragments mapped*.

### Identification and Annotation of DEGs in the Developmental Stages and Different Tissues of *T. multiceps*

**Figure 1:**
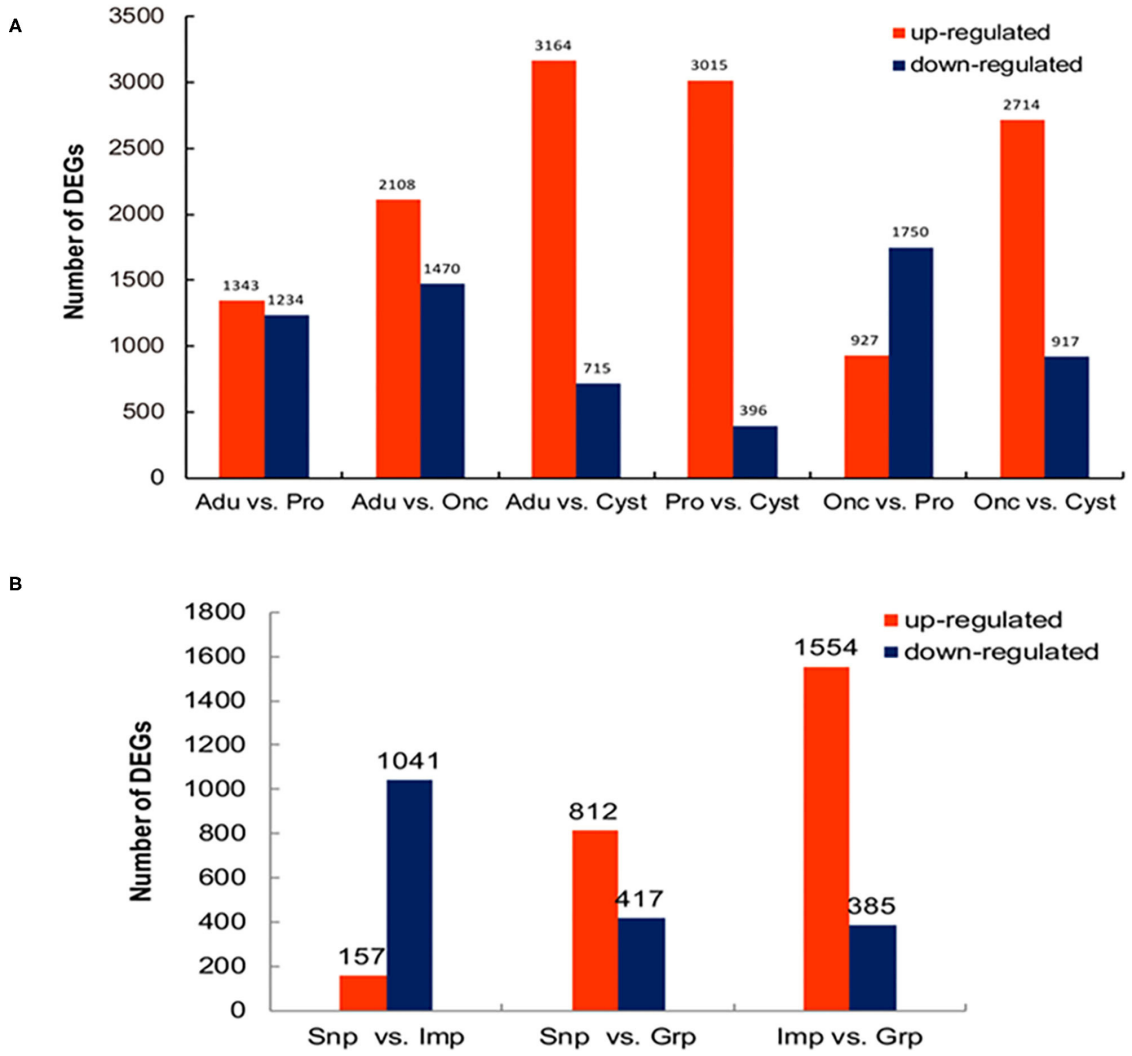
Differentially expressed genes (DEGs) among the developmental stages and tissues of adult *T. multiceps*. **(A)** Numbers of DEGs in the different stages. **(B)** Numbers of DEGs in the different adult tissues.

To comprehensively understand the dynamics of gene expression in *T. multiceps*, we compared the transcriptome data between different life cycle stages and different tissues of adult worms. The total number of DEGs in the various life stages ranged from 2,577 to 3,879; however, for the tissues of the adult worm, the range was from 1,229 to 1,939 (Supplementary Table 1; Figures 1A,B).

**Figure 2:**
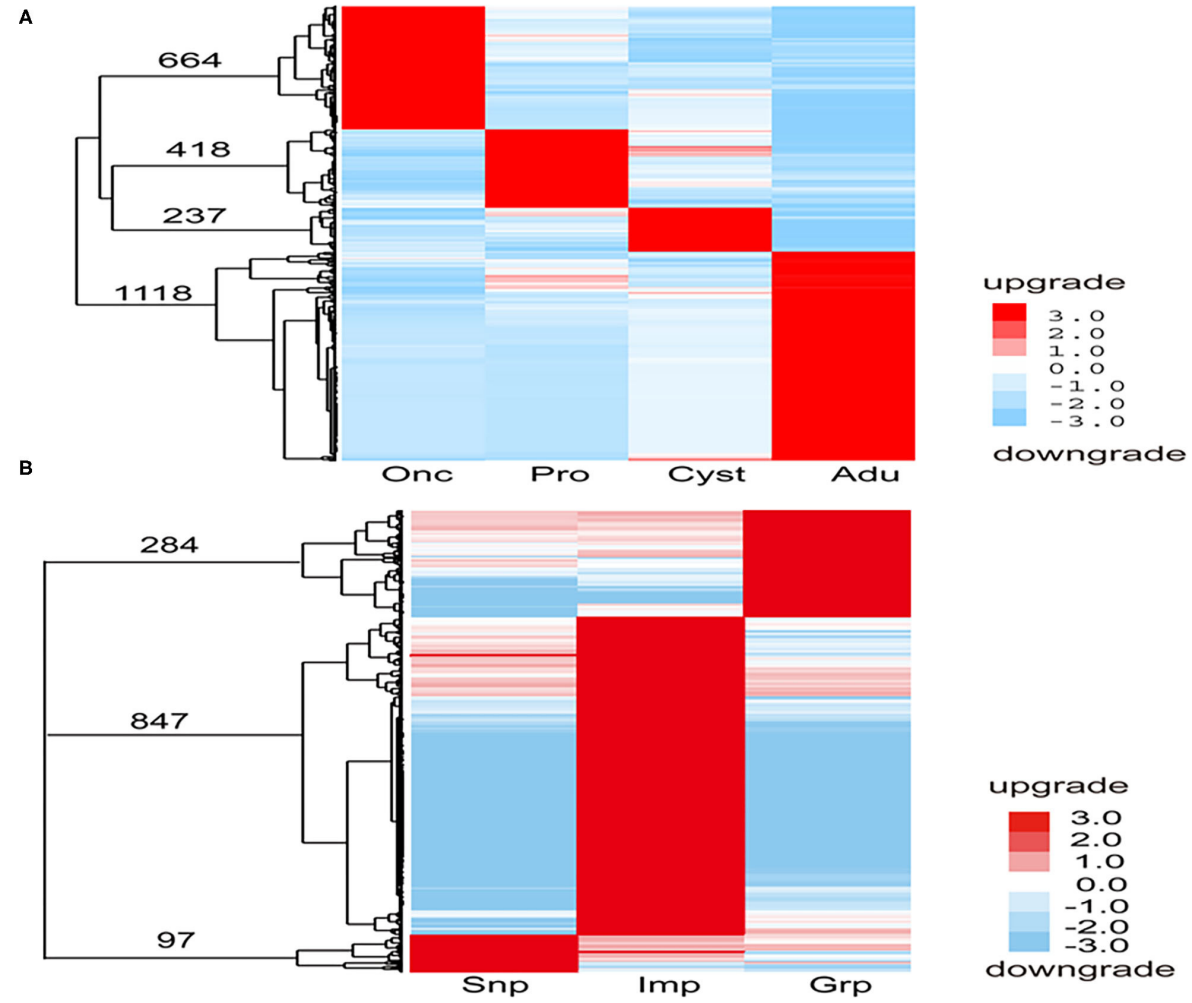
Specific upregulated genes in each development stage and tissue of *T. multiceps*. **(A)** Specific upregulated genes in each developmental stage of *T. multiceps*. **(B)** Specific upregulated genes in Snp, Imp, and Grp of adult *T. multiceps*.

A total of 2,437 DEGs were selected at different developmental stages (Figure 2A). Of these, 664 were oncosphere stage-specific and upregulated, including oncosphere protein Tm18 (Tmu005436), Tm16 (Tmu007899), 45m (Tmu003810), and fibronectin type III domain-containing protein (Tmu011598). Four hundred eighteen of the DEGs were protoscolices stage-specific and upregulated, including immunogenic proteins (Tmu003750), neuronal nitric oxide synthase protein inhibitor (Tmu000145), and inhibitor of apoptosis protein (Tmu004095). In addition, 237 of the DEGs were cyst wall-specific and upregulated, including 8 kDa glycoproteins (Tmu005741, Tmu005823), diagnostic antigen gp50 (Tmu00752), and Hsp90 (Tmu007808). In addition, 1,118 of the DEGs were adult-specific and upregulated, including the tegumental antigen (Tmu000235), ornithine aminotransferase (Tmu000506), heat shock protein 70 (Tmu01113), and tetraspanin (TSP) (Tmu000409). These phase-specific upregulated genes were annotated using GO. Furthermore, there were 456 specific upregulated genes during the oncosphere stage that could be annotated to 31 GO terms. Two hundred thirty-four protoscolices stage-specific upregulated genes were annotated to 32 GO terms, 60 cyst wall-specific upregulated genes to 23 GO terms, and 446 adult-specific upregulated genes to 31 GO terms.

A total of 1,228 DEGs were selected from different developmental tissues (Figure 2B). Ninety-seven of the DEGs were Snp tissue-specific and upregulated, including diagnostic antigens (Tmu006941, Tmu012211, and Tmu012074), TSP (Tmu004996), and profoldin (Tmu010836). Eight hundred forty-seven of the DEGs were Imp tissue-specific and upregulated, including Tmu002740 (similar to ubiquitin/40S ribosomal protein S27a fusion protein) and putative thioredoxin-2 (Tmu000756), whereas 284 of the DEGs were Grp tissue-specific and upregulated, such as the fatty acid-binding protein (FABP) 3 (Tmu003178), insulin-like protein (Tmu004894), epidermal growth factor receptor kinase (Tmu008864), and oncosphere protein Tm18. These tissue-specific upregulated genes were annotated using GO. The 45 Snp tissue-specific upregulated genes were annotated to 21 GO terms, 328 Imp tissue-specific upregulated genes were annotated to 32 GO terms, and 124 Grp tissue-specific upregulated genes were annotated to 27 GO units.

To gain more insight into the specifically upregulated genes in each stage and tissues of the adult *T. multiceps*, the GO database was used to annotate the functions of these specific and upregulated genes. In summary, GO enrichment of the upregulated DEGs in stage-specific and tissue-specific conditions was analyzed. A total of 456 (68.7%), 234 (56.0%), and 446 (39.9%) specifically upregulated genes were assigned to one or more GO categories for Onc, Pro, and Adu, respectively. A total of 45 (46.4%), 328 (38.7%), and 124 (43.7%) specifically upregulated genes were assigned to one or more GO categories for Snp, Imp, and Grp, respectively, as shown in Figures 2A,B. Notably, the number of DEGs among the different stages was higher than that among the different tissues and was consistent with the extent of the phenotypic differences.

**Figure 3:**
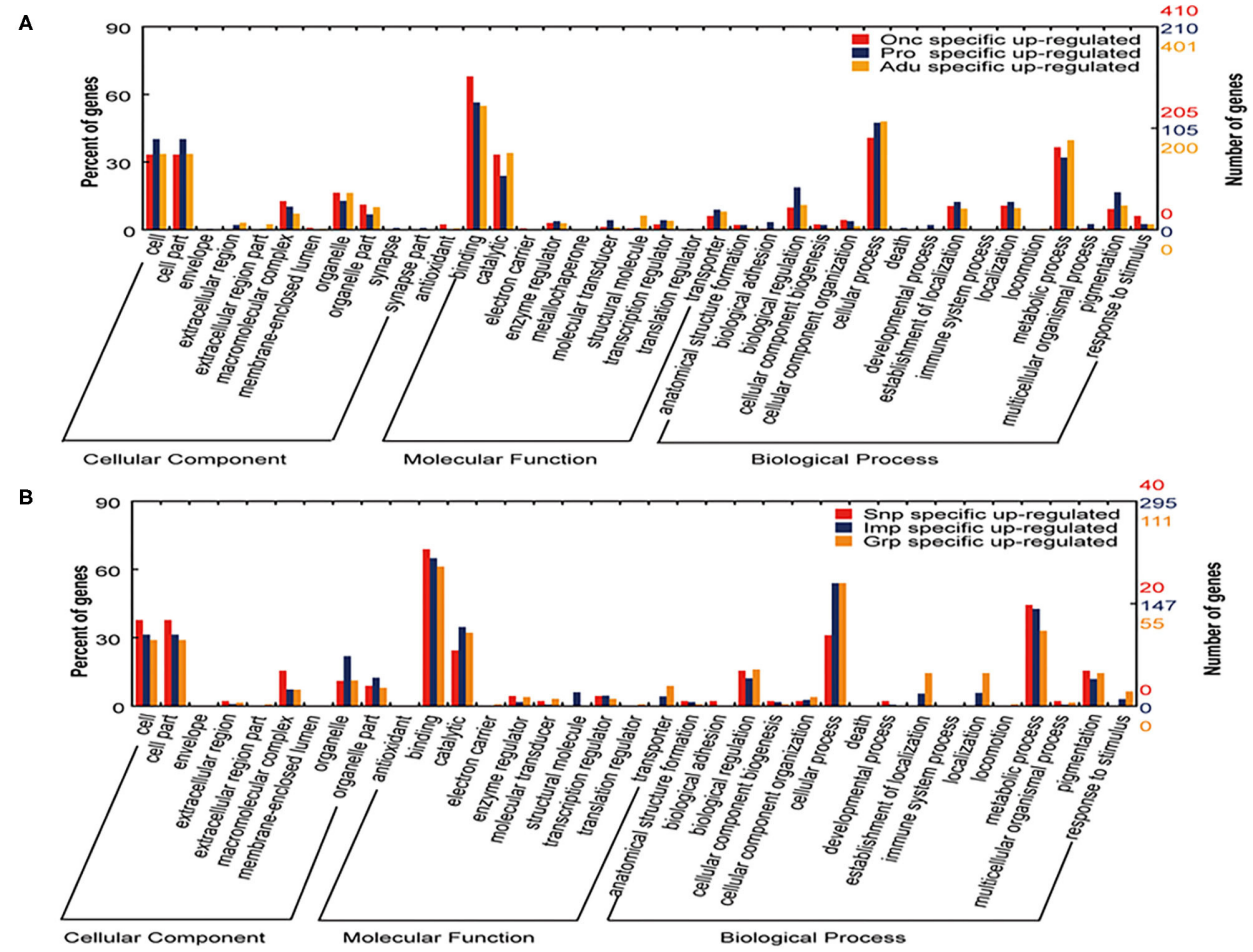
GO analysis of the specific genes upregulated in different developmental stages and tissues of *T. multiceps*. **(A)** Functional classification of specific upregulated genes in three stages (Onc, Pro, and Adu) of *T. multiceps*. **(B)** Functional classification of specific upregulated genes in three tissues of adult *T. multiceps*.

GO classifications enriched in genes for biological process were involved in cellular processes, metabolic processes, pigmentation, and biological regulation; GO classifications enriched in genes for molecular function process were involved in binding, catalytic, and transcription regulation; and GO classifications enriched in genes for the cellular component were involved in the regulation of macromolecular complexes and organelle development (Figure 3). The above observations suggest that the process of development from oncospheres to protoscolices and then to adults involves numerous molecular functions and requires extensive biological processes.

In addition, genes coding for an 8-kDa glycoprotein and the oncosphere proteins Tm16 and Tm18 showed obvious stage-specific variation in expression at different developmental stages of *T. multiceps*. In total, 15 of the 16 genes coding for the 8-kDa glycoprotein were expressed with FPKM > 1 (Supplementary Tables 2, 3).

### DEGs Related to Protease Inhibitor

**Figure 4:**
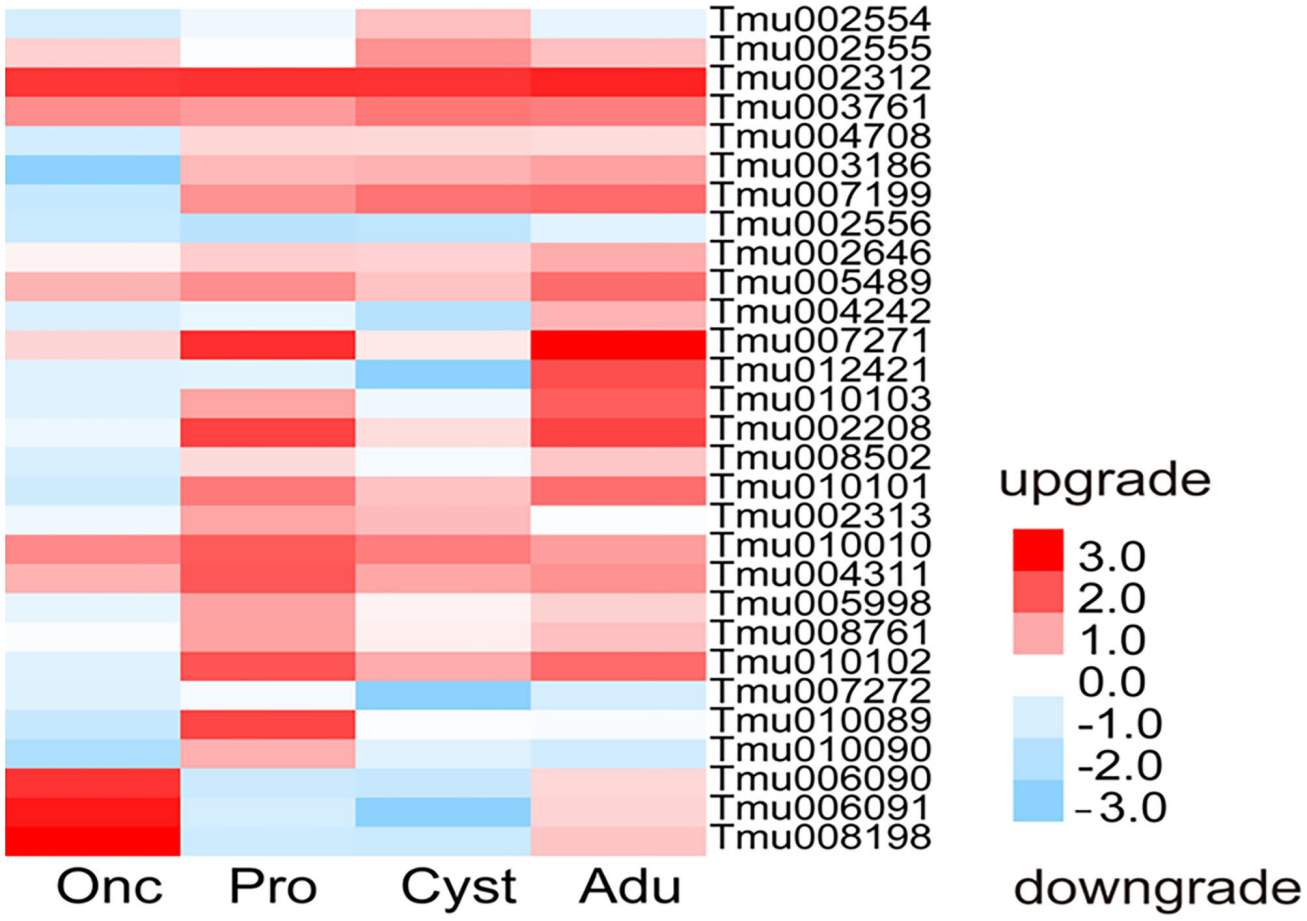
Heatmap of the expression of inhibitor-associated genes in the transcriptomes of *T. multiceps*.

A total of 29 protease inhibitor-associated genes were found in the transcriptomes of *T. multiceps* and consisted mostly of a trypsin inhibitor, followed by serine and cysteine protease inhibitors, with varying expression levels in the different developmental stages. A comparative analysis of the gene expression levels between Adu, Pro, and Onc showed that Tmu006090, Tmu006091, and Tmu008198 were upregulated in Onc; Tmu010089, Tmu010102, Tmu004311, Tmu010010, Tmu008761, Tmu005998, Tmu002313, Tmu010090, and Tmu007272 were upregulated in Pro; whereas Tmu007271, Tmu012421, Tmu010103, and Tmu004242 were upregulated in Adu. However, Tmu002208, Tmu007199, Tmu005489, Tmu010101, Tmu003186, Tmu002646, Tmu008502, and Tmu004708 were highly expressed in Adu and Pro, in contrast to Onc (Figure 4).

KEGG pathway analysis indicated that most of the upregulated DEGs were significantly involved in metabolism, genetic information processing, cellular processes, and environmental information processing, such as glutathione metabolism, nitrogen metabolism, endoplasmic reticulum protein processing, endocytosis, and ubiquitin-mediated proteolysis. Furthermore, upregulated DEGs related to the Wnt signaling pathway, the mitogen-activated protein kinase (MAPK) signaling pathway, and the TGF-β signaling pathway were significantly abundant in the adult stage. Those related to inositol phosphate metabolism, citric acid cycle, and adhesion-related genes were significantly abundant in oncospheres, while progesterone-mediated oocyte maturation and oocyte meiosis-related DEGs were significantly abundant in Imp.

### DEG Confirmation Using Real-Time PCR

**Figure 5:**
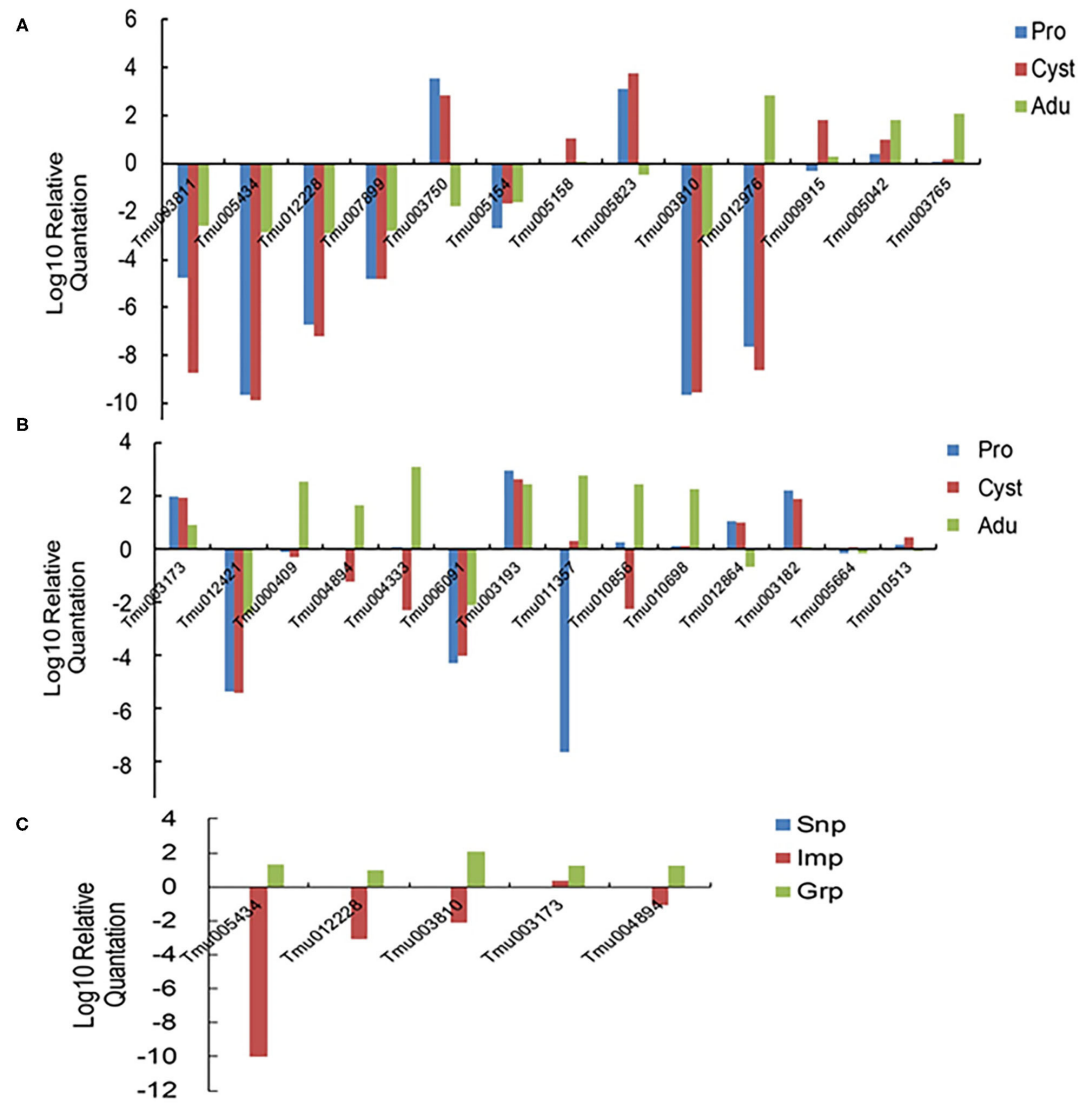
Relative expression levels of the 28 selected DEGs determined using qRT-PCR. **(A,B)** Confirmation of the DEGs in the developmental stages of *T. multiceps*. **(C)** Confirmation of the DEGs in different tissues in the adult stage of *T. multiceps*.

Twenty-eight genes from the DEGs designated Tmu003811 (oncosphere protein Tso31b), Tmu005434 and Tmu012228 (oncosphere protein Tm18), Tmu007899 (oncosphere protein Tm16), Tmu005154 (8 kDa glycoprotein), Tmu003810 (45 m oncosphere protein), Tmu003173 (cytosolic FABP), Tmu000409 (TSP family), Tmu011357 and Tmu012976 (T-Box domain signature), Tmu004894 (insulin-like growth factor), Tmu007712 and Tmu010856 (Unknown), and heat shock protein (Tmu007808) were selected for qRT-PCR analysis to quantify their transcription levels (Figure 5). Of these genes, Tmu003811, Tmu005434, Tmu012228, Tmu007899, Tmu003810, Tmu012421, and Tmu006091 were downregulated in protoscoleces, cyst wall, and the adult stage compared with those in the oncosphere stage, whereas Tmu005042, Tmu003173, and Tmu003193 were upregulated. Meanwhile, Tmu005434, Tmu012228, Tmu003810, and Tmu004894 were upregulated in Grp compared with those in Snp and Imp. The qRT-PCT results of the 28 genes corroborated their RNA-seq results.

## Discussion

The life cycle of *T. multiceps* requires two hosts (dog and sheep). When the final host ingests the mature *Coenurus* cysts, *T. multiceps* reaches maturity in its intestine around 40–42 days. Thereafter, the dog starts to disseminate 3–4 proglottids daily, which could contain almost 37,000 eggs each (30). *T. multiceps* eggs are usually discharged/released/disseminated to the environment through feces from the proglottids (31). When taken up by the intermediate host with contaminated food or water upon reaching the intestine, eggs with hexacanth larvae release oncospheres that penetrate the intestinal wall and migrate toward target organs (usually the brain) through the bloodstream (32). Why oncospheres have a predilection for certain sites, such as muscle, brain, and subcutaneous tissue, is not clear. The larval stage, known as *Coenurus cerebralis*, causes central nervous system disease in sheep, commonly known as coenurosis. The cyst in the brain of sheep is a large, white, translucent structure containing clear fluid with numerous protoscoleces attached to the wall (32). The biology of cestodes is unique in many aspects, especially regarding their unique development, which is difficult to compare to that of other organisms. In the present study, a comprehensive comparative transcriptome analysis of the different developmental stages of *T. multiceps* was carried out for the first time, and 2,437 DEGs were obtained, including oncosphere-specific proteins, immunogenic proteins, diagnostic antigens, heat shock proteins, and tegumental antigens. Podesta and Siddiqui (33) suggested that protein synthesis in flatworm parasites is controlled post-transcriptionally and that these intracellular regulatory mechanisms are activated or suppressed by effectors of the host's immune response. It is evident that this phase-specific or upregulated gene is important for adapting to parasitic hosts and certain sites. This is similar to our previous study, in which the lifecycle process of the oncosphere, larvae, and adults to adapt to the new host environment is a complex differentiation process that is regulated through various protein expressions (16).

Cestodes generally show impressive adaptation to parasitism, including the complete lack of a digestive system, complex life cycle that typically involves two or more hosts, specialized life stages for invasion of each host, specialized structures for attachment (such as an anterior, which usually contains attachment organs, such as grooves or suckers), and serial repetition of reproductive structures (14). A well-adapted scolex, an elongated neck, and a strobila are characteristic of adult *T. multiceps*. The scolex is a holdfast organ armed with four suckers and two circles of rostellar hooks numbered between 18 and 34 (34). The scolex appears to act as an organ of attachment and may also have nutritive and/or sensory functions. In the present study, three of the seven Hsp90 genes (Tmu009603, Tmu007811, and Tmu007806) were upregulated in the adult stage; some reports have suggested that the *Hsp90* gene controls stage differentiation in *Leishmania donovani* (35). Moreover, the *Hsp90* gene has been found to correlate with strobilization in *Echinococcus granulosus* (36). Therefore, we hypothesized that the three *Hsp90* genes in *T. multiceps* correlate with strobilization in adult worm proglottids. Our study also revealed that FABP was upregulated in protoscoleces and the cyst wall. Similar to what we observed, Basika et al. (20) showed that FABP transcripts were abundant in *Mesocestoides corti*. FABP is known to participate in fatty acid transport to obtain essential lipids from the host and to modify them according to the parasite's needs, as cestodes lack the ability to synthesize fatty acids and cholesterol de novo (37). Nie et al. (38) confirmed that FABPs were mainly distributed in the muscle layer in the protoscolices and cells between the body wall and the parenchyma layer of the cestode in various life stages of *T. multiceps*, suggesting that the FABP is closely related to the formation or development of protoscolices and their adaptation to a parasitic environment. Of note, the TSP (Tmu000409) gene was significantly upregulated in adults. TSPs have been used as vaccine candidates against schistosomiasis and echinococcosis and as diagnostic antigens for cysticercosis (39–42). Tran et al. (43) demonstrated that TSPs in the tegument of schistosomula and adult worms can act as receptors for host ligands, including MHC molecules, allowing parasites to mask their non-self-status and escape host immune responses. Furthermore, Huang et al. (44) identified 11 amino acid sequences in the TSP superfamily of *E. multilocularis*, and the Em-TSP1 homologs were found to be highly expressed in early-stage metacestodes compared with those in non-activated and activated oncospheres; however, the expression level of Em-TSP3 varied within non-activated oncosphere samples. In summary, the stage-specific expression of the TSP gene in *T. multiceps* requires further verification.

In the present study, three, nine, and four protease inhibitor-associated genes were upregulated in the oncosphere, protoscolices, and adult stage, respectively. Because tapeworms lack a gut, all nutritive materials must pass through the body surface in the same way as waste materials. The complex cestode tegument contains specific systems for molecular and ion transport, especially amino acids, hexose sugars, vitamins, purines, pyrimidines, nucleotides, and lipids. It might also serve as a major site for catalytic activities and contains enzymes of parasitic and possibly of host origin (45, 46). It is well-known that in these developmental stages, the tapeworm must resist the digestive action of gastric acid in the internal environment of the respective hosts to survive; therefore, the protease inhibitor-associated genes mentioned above may play a critical role in protecting them from the host digestion system.

We surprisingly found a highly expressed 8 kDa glycoprotein in Pro; some information is available concerning 8 kDa as a serodiagnosis antigen in *T. solium* cysticercosis (47). In addition, this 8 kDa glycoprotein from *Echinococcus multilocularis* has been identified as a potential diagnostic marker in patients with alveolar echinonococcosis (48). We predict that the 8 kDa glycoprotein may be outside for the serodiagnosis of *T. multiceps* coenurosis in the future.

Although KEGG pathways involved in metabolism, genetic information processing, cellular processes, and environmental information processing were identified in the present study, unique pathways were observed in the different stages of *T. multiceps*. For instance, genes associated with the Wnt, MAPK, and TGF-β signaling pathways were significantly abundant in the adult stage, which suggested that the growth and development of the adult tapeworm rely on the above signaling pathways as they play key roles in the regulation of cell growth, differentiation, and tissue and organ formation (49, 50).

## Conclusions

In summary, we employed an Illumina RNA-Seq platform to identify DEGs in different developmental stages and tissues of *T. multiceps*. We found that the differential expression of some important genes may contribute to the development of *T. multiceps* and parasite–host interaction.

## Data Availability Statement

The RNAseq data from this study have been deposited in NCBI's Sequence Read Archive and are accessible through accession NumberSRX2357663, SRX2357664, SRX2357665, SRX2357666, SRX2357667, SRX2357668, SRX2357669. https://trace.ncbi.nlm.nih.gov/Traces/sra/?run=SRR5032913.

## Ethics Statement

The animal study was reviewed and approved by the Animal Administration and Ethics Committee of Lanzhou Veterinary Research Institute, Chinese Academy of Agricultural Science. Written informed consent was obtained from the owners for the participation of their animals in this study.

## Author Contributions

BF and WHL conceived and designed the study. WHL wrote the manuscript. YY performed the experiments. NZZ analyzed the data. YJL and JKW assisted to collect samples. LL, HBY, and WZJ helped in the implementation of the study. All authors contributed to the article and approved the submitted version.

## Conflict of Interest

The authors declare that the research was conducted in the absence of any commercial or financial relationships that could be construed as a potential conflict of interest.

## Supplementary Material

Supplementary Table 1 Differentially expressed genes between the developmental stages or tissues.

Supplementary Table 2 Expression of the 8 kDa glycoprotein in the different developmental stages of *T. multiceps*.

Supplementary Table 3 Expression of the oncosphere proteins, Tm16 and Tm18, in *T. multiceps*.
